# Antifibrotic preventive effect of polyethylene glycol (PEG) 3350 in methotrexateinduced hepatoxicity model

**DOI:** 10.1590/acb370507

**Published:** 2022-07-22

**Authors:** Hüseyin Acar, Omay Sorgun, Güner Yurtseve, Ejder Saylav Bora, Oytun Erbaş

**Affiliations:** 1 MD. Izmir Atatürk Training and Research Hospital – Department of Emergency Medicine – Izmir, Turkey.; 2 MD. Ödemiş State Hospital – Department of Emergency Medicine – İzmir, Turkey.; 3 Associate professor. Demiroğlu Bilim University – Faculty of Medicine – Department of Physiology – Istanbul, Turkey.

**Keywords:** Methotrexate, Polyethylene Glycols, Liver, Rats

## Abstract

**Purpose:**

Liver damage caused by drugs and other chemicals accounts for about 5% of all cases. Methotrexate (MTX), a folic acid analogue, is a first-line synthetic antimetabolite agent routinely used in the treatment of rheumatoid arthritis and other autoimmune and chronic inflammatory diseases. Polyethylene glycol (PEG) has antioxidant activity. In this study, we evaluated biochemically and histopathologically the antifibrotic effect of PEG 3350 administered intraperitoneally to prevent methotrexate-induced liver damage in rats.

**Methods:**

A total of 30 male rats including 10 rats was given no drugs (normal group), and 20 rats received single-dose 20 mg/kg MTXfor induced liver injury in this study. MTX was given to 20 rats, which were divided in two groups. Group 1 rats was given PEG30 mg/kg/day (Merck) intraperitoneally, and Group 2 rats % 0.9 NaCl saline 1 mL/kg/day intraperitoneally daily for two weeks.

**Results:**

Transforming growth factor beta (TGF-β), plasma malondialdehyde (MDA), liver MDA, serum tumour necrosis factor alpha (TNF-α), alanine aminotransferase and plasma pentraxin-3 levels and, according to tissue histopathology, hepatocyte necrosis, fibrosis and cellular infiltration were significantly better in MTX+PEG group than in MTX+saline group.

**Conclusions:**

PEG 3350 is a hope for toxic hepatitis due to other causes, since liver damage occurs through oxidative stress and cell damage, similar to all toxic drugs.

## Introduction

The liver is the main organ responsible for drug metabolism and a sensitive target site for substances that modulate biotransformation^1^. Liver damage caused by drugs and other chemicals accounts for about 5% of all cases. These damages have been associated with acute and chronic hepatitis, bile duct abnormalities, neoplasms (oral contraceptives, clomiphene, and carbamazepine), and hepatic adenomas^2^.

Methotrexate (MTX), a folic acid analogue, is a first-line synthetic antimetabolite agent routinely used in the treatment of rheumatoid arthritis and other autoimmune and chronic inflammatory diseases^3^. In diseases such as acute leukaemia and psoriasis, side effects such as progressive hepatic fibrosis and liver damage that can lead to cirrhosis are frequently observed in the case of prolonged MTX treatment. The risk of developing cirrhosis in patients receiving high-dose MTX therapy is 7%^4^. Although gastrointestinal, hepatic, renal and bone marrow toxicities are common side effects, the most serious side effect of MTX is hepatotoxicity^5^. These effects are mostly due to oxidative stress caused by reactive oxygen radicals (ROS)^6^. Many antioxidant agents used can largely eliminate these risks^7^.

Nowadays, many antioxidant agents are used due to their protective and therapeutic properties, and they can largely eliminate the risks of oxidative damage^7^. Polyethylene glycol (PEG) is a safe, affordable, widely available, and highly effective osmotic laxative that acts as a faecal cleaner to remove faecal nitrogen^8^. In addition to its laxative activity, PEG also has antioxidant activity^9^. There are few studies evaluating the effectiveness of antioxidant property in preventing hepatotoxicity, and the results are promising^10^^,^ 11. In cases of acute liver failure due to drugs or chemicals, which are also common in emergency services, effective treatment methods are needed due to the inadequacy of treatment and preventive practices.

In this study, we evaluated biochemically and histopathologically the antifibrotic effect of PEG 3350 administered intraperitoneally to prevent MTX-induced liver damage in rats.

## Methods

This study used 30 male Wistar albino rats, 10-12 weeks old, weighing 150-200 g. The experiments performed in this study have been carried out according to the rules in the Guide for the Care and Use of Laboratory Animals adopted by the National Institutes of Health (United States of America), having received Animal Ethics Committee’s consent (Science University, Ethical number 20210922). The study was carried out in the experimental animal central laboratory of Istanbul Bilim University. Rats were fed *ad libitum* and housed impairs in steel cages in a temperature-controlled environment (22 ± 2°C) with 12-h light/dark cycles.

### Experimental protocol

A total of 30 male rats was used in the study. Among these rats, 10 did not receive any treatment (normal group). The remaining 20 rats were distributed in two groups, including 10 rats treated with saline 1 mL/kg/day intraperitoneally (group 1) and 10 rats treated with PEG 3350 30 mg/kg/day (Merck) intraperitoneally for two weeks (group 2), following administration of a single-dose 20 mg/kg MTXto induce liver injury. Three rats that received saline died during the study. The rats were euthanized, and blood samples were collected by cardiac puncture for biochemical analysis. Liver was removed for histopathological and biochemical examination.

### Histopathological evaluation

Formalin-fixed rectum and colon sections (4 μm) were stained with haematoxylin and eosin. All sections were photographed with Olympus C-5050 digital camera mounted on Olympus BX51 microscope.

Liver histopathological scoring analysis was performed according to Lobenhofer *et al*.^12^. The researcher who performed the histopathological evaluation was blind to the study. The assessment was expressed as the sum of the individual score grades 1 (minimal), 2 (mild), 3 (moderate), and 4 (marked) for each of the following parameters from liver sections: hepatocyte necrosis, fibrosis and cellular infiltration.

### Measurement of plasma tumour necrosis factor-α levels

Plasma tumour necrosis factor-α (TNF-α) levels were measured using commercially available enzyme-linked immunosorbent assay (ELISA) kit (ELK Biotechnology, Wuhan, ELK1396. Detection range: 15.63-1,000 pg/mL). The plasma samples were diluted 1:2, and TNF-α was determined in duplicate according to the manufacturer’s guide. The detection range for TNF-α assay was <2 pg/mL.

### Measurement of plasma alanine aminotransferase levels

Plasma alanine aminotransferase (ALT) levels were measured using commercially available ELISA kit (USCN, Life Science Inc. SEA207Ra. Detection Range: 0.78-50 ng/m).

### Measurement of plasma transforming growth factor beta

Transforming growth factor beta (TGF-β) in plasma was measured using a commercially available ELISA kit (Bioassay Technology, Zhejiang, E0660Mo. Detection range: 5-2,000 pg/mL). TGF-β levels were expressed as pg/mL.

### Measurement of plasma lipid peroxidation

Lipid peroxidation was determined in tissue and plasma samples by measuring malondialdehyde (MDA) levels as thiobarbituric acid reactive substances (TBARS)^13^. Briefly, trichloroacetic acid and TBARS reagent were added to the tissue samples, then mixed and incubated at 100°C for 60 min. After cooling on ice, the samples were centrifuged at 3,000 rpm for 20 min, and the absorbance of the supernatant was read at 535 nm. MDA levels of tissue were calculated from the standard calibration curve using tetraethoxypropane and expressed as nmol/gr protein.

### Measurement of plasma pentraxin-3 level

Plasma pentraxin-3 (PTX3) levels were measured in each 100 μL sample by standard ELISA apparatus at 450 nm by using a PTX3 kit (ELK Biotechnology, Wuhan, ELK6256. Detection range: 31.25-2,000 pg/mL). PTX3 levels were determined in duplicate according to the manufacturer’s guide.

### Statistical analysis

Data analyses were performed using Statistical Package for the Social Sciences (SPSS) version 15.0 for Windows. The groups of parametric variables were compared by Student’s t-test and analysis of variance (ANOVA). The groups of non-parametric variables were compared by Mann-Whitney U test. Results were given as mean ± standard error of mean (SEM). A value of p < 0.05 was accepted as statistically significant, and p < 0.001 was accepted as statistically highly significant.

## Results

A total of 30 male rats was used in the study, of which 10 were in the normal group, 10 in the MTX and saline group, and 10 in the MTX and PEG 3350 group.

**Figure 1:**
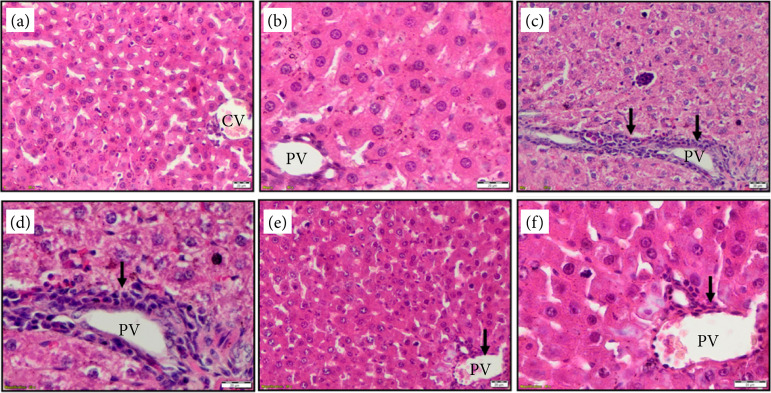
Liver histolopathology haematoxylin and eosin (20× and 40× magnification). (**a** and **b**) Normal liver, central vein (CV) and portal vein (PV); (**c** and **d**) methotrexate and saline groups have bridging necrosis, fibrosis and cellular infiltration in portal area (PV) (*arrow*); (**e** and **f**): methotrexate and polyethylene glycol groups have decreased bridging necrosis fibrosis and cellular infiltration in portal area (PV) (*arrow*).

When comparing normal group and MTX group given with saline, TGF-ß, plasma MDA, liver MDA, serum TNF-α, ALT and plasma PTX3 levels were found to be significantly higher in the MTX group given with saline compared to the normal group (Table 1). In the histopathological examination, no pathological changes were observed in the normal rat group, whereas hepatocyte necrosis, fibrosis and cellular infiltration were observed in the MTX group given with saline (Fig. 1). According to tissue histopathology, hepatocyte necrosis, fibrosis and cellular infiltration were found to be statistically higher in the MTX group given with saline compared to the normal group (Table 2).

When comparing MTX group given with PEG 3350 and MTX group given with saline, TGF-ß, plasma MDA, liver MDA, serum TNF-α, ALT and plasma PTX3 levels were found to be significantly higher in the MTX group given with saline compared to the MTX group given with PEG 3350 (Table 1). In the histopathological examination, hepatocyte necrosis, fibrosis and cellular infiltration were observed in both the MTX group given with saline and the one given with PEG 3350 (Fig. 1). According to tissue histopathology, hepatocyte necrosis, fibrosis and cellular infiltration were found to be statistically higher in the MTX group given with saline compared to the MTX group given with PEG 3350 (Table 2).

**Table 1:** Comparison of biomarker levels between subject groups$.

Biomarker levels	Normal group	MTX and saline group	MTX and PEG group
Plasma TGF-ß (pg/mL)	6.1 ± 1.2	44.7 ± 8.3**	20.7 ± 3.9#
Plasma MDA (nM)	1.03 ± 0.12	2.48 ± 0.07*	1.5 ± 0.2##
Plasma TNF-α (pg/mL)	25.2 ± 4.4	74.1 ± 8.8**	49.7 ± 5.5#
Plasma pentraxin-3 (ng/mL)	1.24 ± 0.2	3.5 ± 0.4*	1.9 ± 0.3##
ALT (IU/L)	47.5 ± 2.3	62.6 ± 1.5*	44.8 ± 1.8#
Liver MDA (nmol/g tissue)	3.5 ± 0.2	5.2 ± 0.2*	2.1 ± 0.2#

$ Results were presented as mean ± standard error of mean. Statistical analyses were performed by one-way analysis of variance;

* p < 0.01;

** p < 0.001 different from normal groups;

# p < 0.05;

## p < 0.001 different from MTX and saline group; MTX: methotrexate; PEG: polyethylene glycol; TGF-ß: transforming growth factor beta; MDA: malondialdehyde; TNF-α: tumour necrosis factor alpha; ALT: alanine aminotransferase.

**Table 2:** Comparison of histopathological findings between subject groups$.

Histopathological finding	Normal group	MTX and saline group	MTX and PEG group
Hepatocyte necrosis	0.16 ± 0.1	2.9 ± 0.4**	0.6 ± 0.1#
Fibrosis	0.3 ± 0.1	2.7 ± 0.5*	0.5 ± 0.2#
Cellular infiltration	0.3 ± 0.1	1.9 ± 0.2*	0.4 ± 0.1#

$ Results were presented as mean ± standard error of mean. Statistical analyses were performed by one-way analysis of variance;

* p < 0.01;

** p < 0.001 (difference from normal group);

# p < 0.05;

## p < 0.001 (difference from MTX and saline group); MTX: methotrexate; PEG: polyethylene glycol.

## Discussion

Drug-induced hepatotoxicity is the most common cause of acute liver failure^14^. One of these drugs causing hepatotoxicity is MTX. Especially in the presence of type-2 diabetes, obesity and alcohol use, the risk of developing liver damage and liver failure increases in patients using long-term methotrexate^15^^-^ 17.

In the clinical diagnosis of liver injury, liver enzymes, many cytokines released in response to liver injury^16^^,^ 18, and some acute phase reactants can be used as biomarkers^19^. It has been shown both experimentally and clinically that the levels of ALT, which are among these biomarkers, increase due to the use of MTX^20^. TGF-β^21^, which is a factor released from the first injury to the liver at all stages of liver diseases, such as in cirrhosis and hepatocellular carcinoma, and TNF-α^22^^,^ 23, an inflammatory cytokine, are other biomarkers whose blood levels increase in liver damage. Cure *et al*.^24^ showed that MTX given to rats in their study significantly increased TNF-α and TGF-β levels. In this study, in accordance with the literature, it was observed that serum ALT, TNF-α, TGF-β levels, demonstrating hepatic injury, increased significantly in the subject group given MTX and saline compared to normal group.

MDA is the end product of lipid peroxidation and a biomarker that indicates oxidative damage^25^. Jahovic *et al*.^26^, in a study with rats, showed that MTX caused an increase in MDA in the blood, liver and kidney. PTX-3, on the other hand, is an acute phase response protein from the catabolite repressor protein family, which is mostly produced by hepatocytes in response to inflammation^19^. However, there are few studies showing the effect of MTX on PTX-3 levels in the blood. Deyab *et al*.^27^ suggested that there was no significant increase in PTX-3 level in romatoid artrit (RA) patients using MTX for six months. In this study, in accordance with the literature, it was observed there was a significant increase in the level of plasma MDA and liver MDA, the biomarkers showing oxidative stress, in the subject group given MTX and saline compared to normal group. Contrary to the study of Deyab *et al*.^27^, the level of serum PTX, another biomarker showing oxidative stress, was found to increase due to the use of MTX. Since Deyab *et al*.^27^ study is clinical, there may be some other reasons that might affect drug metabolism and PTX level. More experimental and clinical studies are needed.

PEG 3350 is an osmotic laxative frequently used in the treatment of constipation^28^^-^ 30. Apart from the treatment of constipation, there are studies showing that it has been tried in the treatment of hepatic encephalopathy due to its faecal nitrogen removal feature and that it is superior to lactulose in this area^31^^,^ 32. There are also studies showing that PEG 3350 prevents oxidative damage^33^^,^ 34. Patlolla *et al*.^35^, in a study investigating the efficacy of PEG to reduce the toxic effects of gold-based nanoparticles (GNPs) on the liver, the coating of GNPs with PEG significantly reduced oxidative stress, showed that AST, ALT and ALP values were significantly lower than the PEG uncoated GNPs group. As far as we know, there is no study in the literature investigating the effect of PEG on MTX-induced hepatotoxicity. In this unique study, it was observed that the ALT, TNF-α, TGF-β, plasma MDA, liver MDA, and PTX-3 levels of the subject group given MTX and PEG 3350 were significantly lower than the group given MTX and saline. In this unique study, levels of ALT, TNF-α, and TGF-β, which indicate liver damage, as well as levels of plasma MDA, liver MDA and PTX-3, which indicate oxidative stress, were found to be significantly lower in the PEG group compared to the saline group. These results can be interpreted as PEG 3350 has antioxidant potential and prevents MTX-induced hepatotoxicity.

Drug-induced liver damage and hepatotoxicity are frequently seen conditions that can be fatal if left untreated^36^^,^ 37. MTX, which is used in many diseases such as RA and psoriasis, is one of the hepatotoxic drugs known to cause hepatocyte necrosis, fibrosis, and cellular infiltration in the liver^38^^,^ 39. There are many drugs used to prevent the toxic effects of MTX^40^^-^ 42.However, there are very few studies in which the histopathological evaluation of the inhibitor effect of PEG 3350 on MTX induced liver damage^43^. Patlolla *et al*.^35^, in a study in which they evaluated the effect of PEG coated GNPs on liver toxicity histopathologically, reported that GNPs significantly reduced damage on hepatocytes and central veins. In the histopathological examination performed in this study, severe bridging necrosis, fibrosis and cellular infiltration were observed in the portal area in the group given MTX and saline. In the group given MTX and PEG 3350, the incidence of bridging necrosis, fibrosis and cellular infiltration in the portal area was significantly lower. These results histopathologically suggest that PEG 3350 prevents MTX-induced hepatotoxicity.

Studies investigating the use of PEG in the prevention of liver toxicity are limited to animal experiments, and there is no similar study in humans. Therefore, we do not have enough information about the clinical use of PEG yet. Further studies with humans may clarify this issue. However, PEG, which is used as a laxative in humans, is only administered orally and excreted without being absorbed. Therefore, PEG administered orally does not show any systemic effect. In humans, it is not known whether the intraperitoneal route or the intravenous route can be used, which may be a possible limitation in clinical studies.

## Conclusions

In this study, it was seen that ethylene glycol administered intraperitoneally prevented the negative effects of MTX on liver damage in rats. We believe that PEG 3350 is also a hope for toxic hepatitis due to other causes. Further studies will clarify this hypothesis.
